# Dysphagia: A Rare Presentation of Sarcoidosis

**DOI:** 10.7759/cureus.19397

**Published:** 2021-11-09

**Authors:** Baha Aldeen Bani Fawwaz, Aimen Farooq, Ahmad Al-Dwairy, Akram I Ahmad, Arooj Mian, Abu H Khan

**Affiliations:** 1 Internal Medicine, AdventHealth Orlando, Orlando, USA; 2 Internal Medicine, MedStar Washington Hospital Center, Washington, USA; 3 Gastroenterology and Hepatology, AdventHealth Orlando, Orlando, USA

**Keywords:** esophageal sarcoidosis, gi sacrcoidosis, esophagus, sarcoidosis, dysphagia

## Abstract

Gastrointestinal (GI) sarcoidosis is a rare manifestation of this multi-systemic granulomatous disorder. Esophageal involvement is extremely rare and there have been few case reports about this. Our article reports a case of esophageal sarcoidosis in which dysphagia was the main presenting symptom. The main initial treatment of symptomatic sarcoidosis in general and pulmonary sarcoidosis in specific usually involves corticosteroids, however, there are no specific guidelines for the management of GI sarcoidosis. Surprisingly, or maybe not, in our case, the dysphagia did not improve with steroid therapy which prompted further investigations as well as endoscopic intervention.

## Introduction

Sarcoidosis is a multi-systemic inflammatory disease that affects different races but is more commonly seen in African Americans with an estimated annual incidence of 35.5 per 100,000 in the aforementioned population. Although the exact etiology of the disease is still unidentified, it is well known that the presence of non-caseating granulomas is the main pathological finding in the affected organs [1]. Up to 60% of sarcoidosis cases are asymptomatic and incidentally discovered after routine chest film. Among symptomatic patients, respiratory and constitutional symptoms are the most common presenting symptoms [2]. Extra-pulmonary involvement of sarcoidosis is common and has been reported in a variety of organs including the eyes, liver, heart, nervous system, and kidneys [3]. However, extra-hepatic gastrointestinal (GI) involvement is uncommon [4,5]. We present a case of sarcoidosis with esophageal involvement in which dysphagia was the main presenting symptom.

## Case presentation

A 32-year-old male patient with no significant past medical history other than COVID-19 infection, months prior to admission, presented to ED complaining of dysphagia. Symptoms began about 1 week prior to presentation with difficulty swallowing liquids that progressed to involve solids as well. Dysphagia was described by the patient as a choking sensation and that he feels the food getting stuck in his chest. Dysphagia is partially relieved with belching. The patient also reported pyrosis and occasional vomiting. Denied nausea, abdominal pain or any change in bowel habits. On review of systems, the patient endorsed subacute cough for 3-4 weeks duration. The cough was mainly nonproductive and has been worsening since onset. It was associated with shortness of breath. Shortness of breath occurred mainly with exertion and while talking. The patient denied hemoptysis, fevers, chills, night sweats, weight loss, myalgia and arthralgia. 

On presentation, the patient was afebrile, HR: 94, RR: 19, O_2_sat: 100% RA and BP: 129/81. Examination revealed mild wheezes over lung bases bilaterally and no palpable lymphadenopathy. The remainder of the physical examination was unremarkable. Complete blood count and comprehensive metabolic panel were within normal range. Angiotensin-converting enzyme level was elevated at 81 U/L (Normal range 9 - 67 U/L). HIV Ag/Ab screening test was negative as well as COVID-19 PCR test. 

**Figure 1:**
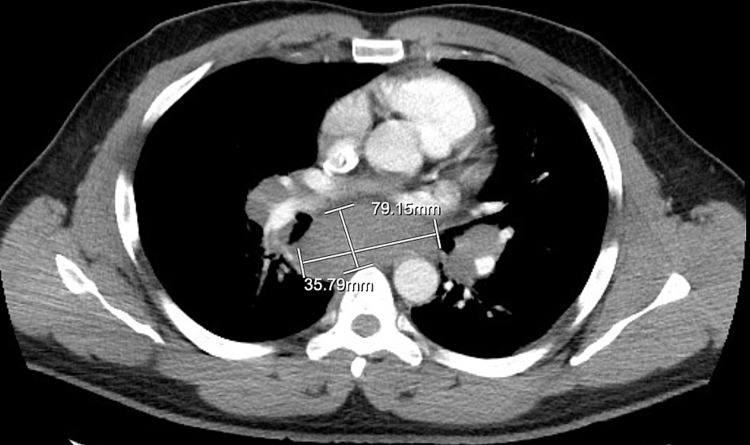
CT chest without contrast Multiple arch mediastinal and bilateral hilar lymph nodes, including a subcarinal lymph node conglomerate measuring 7.9 x 3.6 cm.

**Figure 2:**
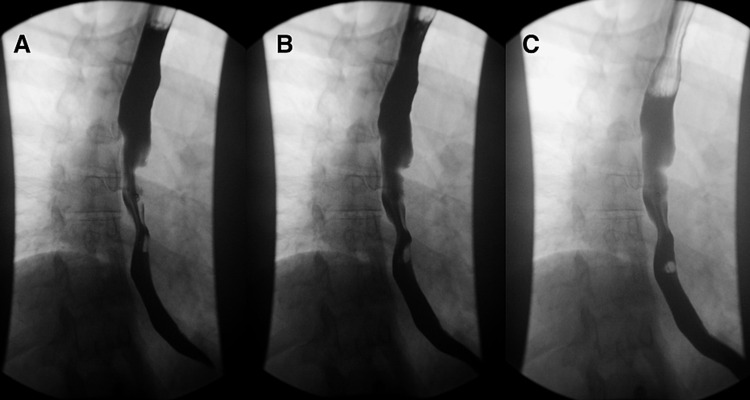
Barium esophagram (A-C) Extrinsic mass affect involving the middle esophagus with no evidence of stricture.

CT-chest with contrast (Figure 1) showed prominent mediastinal and bilateral hilar adenopathy, multiple pulmonary nodules, mild interlobular septal thickening, suggesting interstitial pulmonary edema and peripheral left lower lobe ground-glass opacities, which could be pulmonary edema or infection. Esophagram (Figure 2) showed findings compatible with extrinsic mass effect involving the middle esophagus, in keeping with bulky mediastinal lymphadenopathy noted on CT chest.

On the third day of admission, the patient underwent Endobronchial Ultrasound Bronchoscopy (EBUS) with transbronchial needle aspiration. Samples were sent for cytology, pathology and flow cytometry. Flow cytometry was negative for monoclonal B-cells and immuno-phenotypically abnormal T-cells. The pathology report showed abundant non-necrotizing granulomas, mature lymphocytes and endobronchial cells in all biopsied samples. Grocott methenamine silver (GMS) and AFB stains were negative for microorganisms. Fungus and AFB cultures were negative as well.

The patient was tolerating PO intake and was discharged from the hospital. The patient followed up with the pulmonology clinic one week after discharge for biopsy results and was started on oral prednisone 40 mg qDay. The patient respiratory symptoms did improve after three months of steroid therapy. Nonetheless, dysphagia persisted. Due to the persistence of dysphagia and as evidence of esophageal compression does not rule out infiltrative involvement. The patient underwent EGD with dilation followed by manometry studies a few months after discharge. The mucosa was grossly and histologically normal. Manometry studies were also unremarkable. The patient reported partial improvement of dysphagia after dilation. The patient is still following up with pulmonology and gastroenterology clinics for further evaluation and management.

## Discussion

Digestive tract sarcoidosis is rare [5]. In one case series, esophageal sarcoidosis was reported in about 14% of GI sarcoidosis cases. In those cases, dysphagia was the main presenting symptom [4]. Mechanisms by which sarcoidosis can affect the esophagus can be divided mainly into infiltration and extrinsic compression. Inflammatory Infiltration can involve mucosa, muscular layer or myenteric plexus. That inflammatory infiltrates can form strictures and/or nodules in case of mucosal involvement, while muscular and myenteric plexus involvement lead to malfunctioning of the affected part resulting in dysphagia. Extrinsic compression is believed to be mainly related to mediastinal lymphadenopathy, which is a common finding in sarcoidosis [6]. This adenopathy can lead to direct compression, such in our case, or it can also lead to traction esophageal diverticulum or even perforation [4].

The diagnostic approach for dysphagia includes performing EGD or esophagram depending on whether oropharyngeal involvement is present or not. In our case, the patient's description for dysphagia was not oropharyngeal, i.e., the patient had no difficulty initiating swallowing and felt the food getting stuck at chest/mid esophagus level. However, CT chest findings of prominent mediastinal adenopathy have sparked the clinical suspicion of compressive adenopathy etiology which did correlate with mid esophagus level. Hence, we decided to start with esophagram.

Management of compressive dysphagia focuses mainly on relieving the compressive etiology. In our case, it was the inflammatory adenopathy; thus, the patient was started on corticosteroid therapy. Patient symptoms did not improve with steroids which prompted further management with endoscopic dilation. One other temporary option that can be considered is esophageal stenting. In case of failure to respond to steroids, surgical intervention might also be an option.

## Conclusions

Sarcoidosis is an inflammatory disease that can involve the GI tract. Main presenting symptoms of sarcoidosis vary and might include non-respiratory and non-constitutional symptoms. Esophageal sarcoidosis is rare and usually presents with dysphagia. Initial diagnostic approach after confirming sarcoidosis diagnosis includes EGD and/or esophagram. Cornerstone treatment of sarcoidosis in general and GI sarcoidosis remains to include corticosteroids therapy.
